# The RhoGAP Activity of Myosin IXB Is Critical for Osteoclast Podosome Patterning, Motility, and Resorptive Capacity

**DOI:** 10.1371/journal.pone.0087402

**Published:** 2014-01-23

**Authors:** Brooke K. McMichael, Katharine F. Scherer, Nicole C. Franklin, Beth S. Lee

**Affiliations:** Department of Physiology and Cell Biology, The Ohio State University College of Medicine, Columbus, Ohio, United States of America; Inserm U606 and University Paris Diderot, France

## Abstract

Osteoclasts are large, multinucleated cells of the monocyte-macrophage lineage that generate specialized substrate adhesion complexes to facilitate their function as bone-degrading cells. The patterning and function of these actin-based complexes, podosomes and sealing zones, are regulated by the small GTPase Rho. Myosin IXB (Myo9b) is a unique actin-based motor protein that contains a RhoGAP domain, which, like other RhoGAPs, is inhibitory to Rho signaling. In this study, Myo9b is shown to be expressed in osteoclasts and act as a critical regulator of podosome patterning and osteoclast function. SiRNA-mediated knockdown of Myo9b results in increased activity of Rho but not Rac in osteoclasts. Knockdown in osteoclasts on glass results in altered podosome patterning and decreased motility, and this effect is reversed by addition of a Rho inhibitor. SiRNA-mediated suppression of Myo9b expression in osteoclasts on bone results in a dramatic loss of resorptive capacity even though sealing zones appear normal. This loss of resorption is also reversible with addition of a Rho inhibitor. Cells with diminished Myo9b levels display mislocalization and suppressed activation of Src, a tyrosine kinase with critical effects on osteoclast actin cytoskeletal rearrangement and function. In addition, siRNA-treated cells display poorly formed microtubule networks and a lack of tubulin acetylation, a marker of microtubule stability. However, short-term addition of TNFα to cells with suppressed Myo9b levels overcomes or circumvents these defects and causes increased sealing zone size and resorptive capacity. These results indicate that the RhoGAP activity of Myo9b plays a key role in regulating the actin-based structures necessary for osteoclast motility and resorption, and confirms that Myo9b can act as a motorized signaling molecule that links Rho signaling to the dynamic actin cytoskeleton.

## Introduction

Myosins are a large superfamily of actin-based molecular motors involved in a wide variety of cellular functions that include organellar and molecular transport, mitosis and cytokinesis, motility, signal transduction, and maintenance of cell shape [1], [2], [3]. Myosin heavy chains are composed of a head domain that binds actin in an ATP-dependent manner, a neck domain with one or more light chain binding sites (IQ motifs), and a variety of specialized tail domains. The myosin superfamily can be divided into 35 classes, of which 40 different proteins can be found in human and mouse tissues [4].

The mammalian myosin class IX consists of two members, Myo9a and Myo9b. Myo9a is the ortholog of rat myr7 and is expressed primarily in brain, testis, and spleen [5], while Myo9b (rat myr5) is most highly expressed in leukocytes, particularly of myelocytic origin [6]. Lower levels of Myo9b are expressed in some epithelial cells, most notably those of the digestive tract [6], [7]. Indeed, polymorphisms of human *MYO9B* have been associated with inflammatory diseases of the bowel including celiac disease, ulcerative colitis, and Crohn's disease [8], [9], [10], [11], [12]. These intestinal disorders may be due to loss of Myo9b function leading to impaired integrity of epithelial cells in the digestive tract [10], [13], [14]. Class IX myosins are plus-end directed motors, and despite containing a single actin-binding head structure, are also processive [15], [16], [17]. While all other known processive myosins are double-headed and stay attached to the actin filament by walking in a hand-over-hand mechanism [18], single-headed class IX myosins have the capacity to remain attached to actin through a large extended loop in the head domain that prevents dissociation [19]. These myosins also contain N-terminal extensions with similarities to Ras-association domains [6], [20]. The mammalian class IX myosins contain 4–6 IQ motifs in their neck regions, while the tail also contains an atypical C1 domain of unknown function [6], [20]. Finally, class IX myosins contain a RhoGAP (Rho GTPase-activating protein) domain in their tails [5], [20], [21]. This domain, like other RhoGAPs, accelerates hydrolysis of Rho-bound GTP, thereby switching Rho from an active, GTP-bound conformation to an inactive, GDP-bound state. Accordingly, class IX myosins can be considered as “motorized signaling molecules” that can directly link Rho signaling to the actin cytoskeleton [22]. Because Rho regulates actin organization and dynamics, it is feasible that class IX myosins modulate the structure of their actin tracks through various feedback mechanisms as they move along the filaments.

Osteoclasts are large multinucleated cells derived from the monocyte-macrophage lineage that generate attachment structures dependent on the nanotopography and adhesiveness of the substrate [23], [24]. When cultured on smooth surfaces such as glass and plastic, osteoclasts attach to the substrate through podosomes, specialized integrin-based complexes typified by a short F-actin core that rapidly turns over, allowing for a high degree of motility [25]. The arrangement of podosome superstructures changes as osteoclasts mature, starting as clusters that develop into small rings and finally encircle the cell periphery in podosome belts [26]. On mineralized substrates (e.g. bone), osteoclasts form sealing zones, actin-rich ring structures that resemble densely packed podosomes with extensive actin cross-bridges [27]. These sealing zones encircle a specialized secretory apical membrane (the ruffled border) and permit tight attachment of the cell to the substrate. Secretion of proteases (most notably, cathepsin K) and protons from the ruffled border onto the bone surface causes degradation of the collagen matrix and dissolution of hydroxyapatite mineral [28]. In this manner, osteoclasts degrade, or resorb, bone in response to various external signals.

Rho is a critical regulator of actin organization such as stress fibers and focal adhesions. In osteoclasts, Rho is required to maintain podosome and sealing zone organization. Treatment of osteoclasts with exoenzyme C3, which inhibits RhoA, RhoB, and RhoC, causes disruption of the sealing zone [29]. Conversely, activation of RhoA does not stimulate formation of podosomes, but rather causes changes in their distribution [30], [31]. The organizational effects produced by activation of RhoA differ depending on whether osteoclasts are seeded on glass or a bone-like substrate. On glass, RhoA activation drives podosomes into rings and clusters, while on bone, RhoA stimulates sealing zone formation [32], [33], [34]. When RhoA activity is minimal on either substrate, peripheral podosome belts tend to be formed.

Inflammatory diseases of the bowel are commonly associated with poor skeletal health, including growth retardation, osteopenia, and osteoporosis [35], [36]. Because the *MYO9B* gene is associated with these diseases and is a regulator of Rho signaling, we have investigated the role of Myo9b in the morphology, migration, and resorptive ability of osteoclasts. RNA interference studies were performed to determine how alterations in expression of Myo9b might alter podosome and sealing zone patterning, and overall osteoclast activity. These studies demonstrate that the RhoGAP domain of Myo9b plays a key role in osteoclast function through regulating the distribution and stability of cytoskeletal-associated proteins.

## Materials and Methods

### Ethics Statement

Animal studies were approved by University Laboratory Animal Resources at The Ohio State University (protocol number 2007A0175).

### Cell culture, immunocytochemistry, and microscopy

Osteoclasts were generated either from murine bone marrow or the murine macrophage cell line RAW264.7 as previously described [37], [38], [39]. Marrow cells from Swiss-Webster mice, 4–8 weeks in age, were incubated in α-MEM containing 10% fetal bovine serum and supplemented with 20 ng/ml M-CSF (R & D Systems), and 100 ng/ml of a GST-RANKL fusion protein that was previously described [40]. For RAW264.7-derived osteoclasts, the macrophage cell line was cultured in Dulbecco's modified Eagle's medium with 10% fetal bovine serum; differentiation was induced by addition and continued presence of 100 ng/ml GST-RANKL. For both cell types, osteoclasts were assayed at maturity (5–7 days with RANKL). TNFα stimulation of osteoclasts was performed by incubating mature cells overnight in differentiation medium plus 10 ng/ml recombinant TNFα (R & D Systems). Src Inhibitor-1 (a dual site inhibitor of Src kinase) was purchased from Sigma and, where indicated, was added to cells for 1 hour at 44 nM.

Osteoclasts were cultured either on glass coverslips or thinly-cut ivory slices. Following differentiation, cells were fixed, permeabilized, and allowed to bind antibodies as previously described [41], [42]. Primary antibodies were diluted in a blocking buffer composed of 20% fetal bone serum and 1% polyethylene glycol in phosphate-buffered saline, and were detected using Alexa-labeled secondary antibodies (Invitrogen Corp.). F-actin was detected with the use of Alexa-labeled phalloidin (Invitrogen Corp.). DAPI was included in the cell mounting buffer ProLong Gold (Invitrogen Corp.) to identify nuclei. Cells were visualized using an Olympus FV1000 Filter Confocal microscope (Campus Microscopy and Imaging Facility, The Ohio State University). Osteoclasts were identified as cells containing three or more nuclei and staining positive for tartrate-resistant acid phosphatase. Osteoclasts were considered to have reached maturity when they demonstrated a continuous belt of podosomes at the cell periphery [26].

Apoptosis was measured using the In Situ Cell Death Detection Kit (Roche) according to manufacturer's specifications. Measurements of osteoclast and sealing zone size were made using SigmaScan Pro 5.0 software (SPSS Science) and Olympus confocal microscope software.

### RNAi-mediated knockdown of Myo9b

SiRNAs were designed and synthesized by Ambion. SiRNAs 156914, 156915, 156916 were successfully used to produce RNA and protein knock-down and are referred to in this study as siRNAs 14, 15, and 16, respectively. SiRNA transfections into osteoclasts were previously described [37], [39], [41], [42], [43]. On day 4 of osteoclast differentiation, 50 nM of siRNA or an equal concentration of a control siRNA was electroporated at 250 V/50 µF with the siRNA solution or an equal concentration of a control siRNA. For immunocytochemical analysis, the cells were replated on ivory slices or glass coverslips immediately following the transfection. For RNA analysis, total cellular RNA was harvested 1–3 days after the transfection with RNA-Bee (Tel-test, Inc). For protein analysis, whole cell lysates were harvested 1–4 days post transfection with M-PER (Pierce Biotechnology). Optimal protein knockdown was achieved 3 days post-transfection.

Myo9b mRNA levels were assessed both by real-time PCR and by a gel-based competitive RT-PCR. Quantitative real-time PCR was performed using Bio-Rad iCycler technology, with iQ SYBR Green Supermix and Bio-Rad PCR primers for mouse Myo9b and a β-actin standard. Gel-based competitive RT-PCR was also used to visualize mRNA levels. Briefly, a synthetic mRNA internal standard was created that corresponded to the expected Myo9b PCR product, but contained an internal deletion of 26%, a T7 promoter element, and a tail of 15 adenosines, as previously described for other mRNAs [41], [42], [43]. This product was transcribed *in vitro* using the MAXIscript system (Ambion), and 1 picogram of the resulting RNA (the internal standard) was added to 1 µg of osteoclast total cellular RNA prior to reverse transcription and PCR. These reactions were performed using the Superscript First-strand Synthesis System from Invitrogen. The resulting RT-PCR products were run in a 2% gel and stained with ethidium bromide to visualize the relative intensities of the bands, which were measured using Quantity One software (Bio-Rad).

### Myo9b plasmids

A cDNA encoding the tail region of Myo9b, extending from the end of the IQ regions to the C-terminus, was subcloned into expression vector pEF6/V5-His (Invitrogen) and transfected by electroporation into RAW264.7 cells. Stably transfected clones were selected with 3 µg/ml blasticidin.

### Western analysis and small GTPase activity assays

A rabbit polyclonal antibody from Protein Tech group and a goat polyclonal Ab from Imgenex were used to detect Myo9b. Mouse monoclonal antibodies against α-tubulin and acetylated α-tubulin were purchased from Sigma while mouse monoclonal antibodies against total Src and phospho-Src kinase (Tyr416) were purchased from Upstate/Millipore. An antibody that detects phosphotyrosine residues was from BD Transduction Laboratories. Antibodies to loading controls β-actin and GAPDH were purchased from Abcam. Secondary antibodies for chemiluminescent detection were obtained from Jackson Laboratories. PVDF membrane was purchased from GE Healthcare and SuperSignal West Pico detection reagents were purchased from Thermo Scientific.

Rho and Rac activity were measured with protein pull-down Activation Assay kits from Cytoskeleton Inc. Total Rho/Rac levels were compared to active Rho/Rac and measured using Bio-Rad Quantity One software. Rho Inhibitor I (a cell permeant exoenzyme C3 transferase) was purchased from Cytoskeleton Inc.

### Osteoclast Functional Assays

Directional motility was measured by the use of 8.0 µm pore Transwell migration chambers (Corning Life Sciences, Acton, MA). Immediately following transfection, cells were scraped and replated at a low density on the upper side of the chamber. After 3 days, the cells were stimulated to migrate by the addition of an RGD-containing osteopontin peptide to the bottom of the well. After an overnight incubation, cells on the upper side of the membrane were removed with a cotton swab, and the migrated cells were fixed and stained for tartrate resistant acid phosphatase using a Leukocyte Acid Phosphatase kit (Sigma).

Osteoclast resorption assays were begun four days after initial RANKL stimulation. Osteoclasts were transfected and immediately plated on BD BioCoat Osteologic Discs (BD Biosciences) or thinly cut ivory slices as a bone substrate. Control and siRNA-treated cells were kept on the discs/bone for 3 days. The cells were removed by the addition of bleach for 5 minutes and several washes with water or by scraping. Bone was stained 5 minutes with acid hematoxylin, mounted on glass slides, and resorption was assayed by confocal microscopy. BD discs were assayed by photographing under low magnification, and quantifying resorbed areas with SigmaScan Pro 5.0 software (SPSS Science). Equal numbers of images were compared among test groups.

## Results

### Distribution of Myo9b in osteoclasts

**Figure 1:**
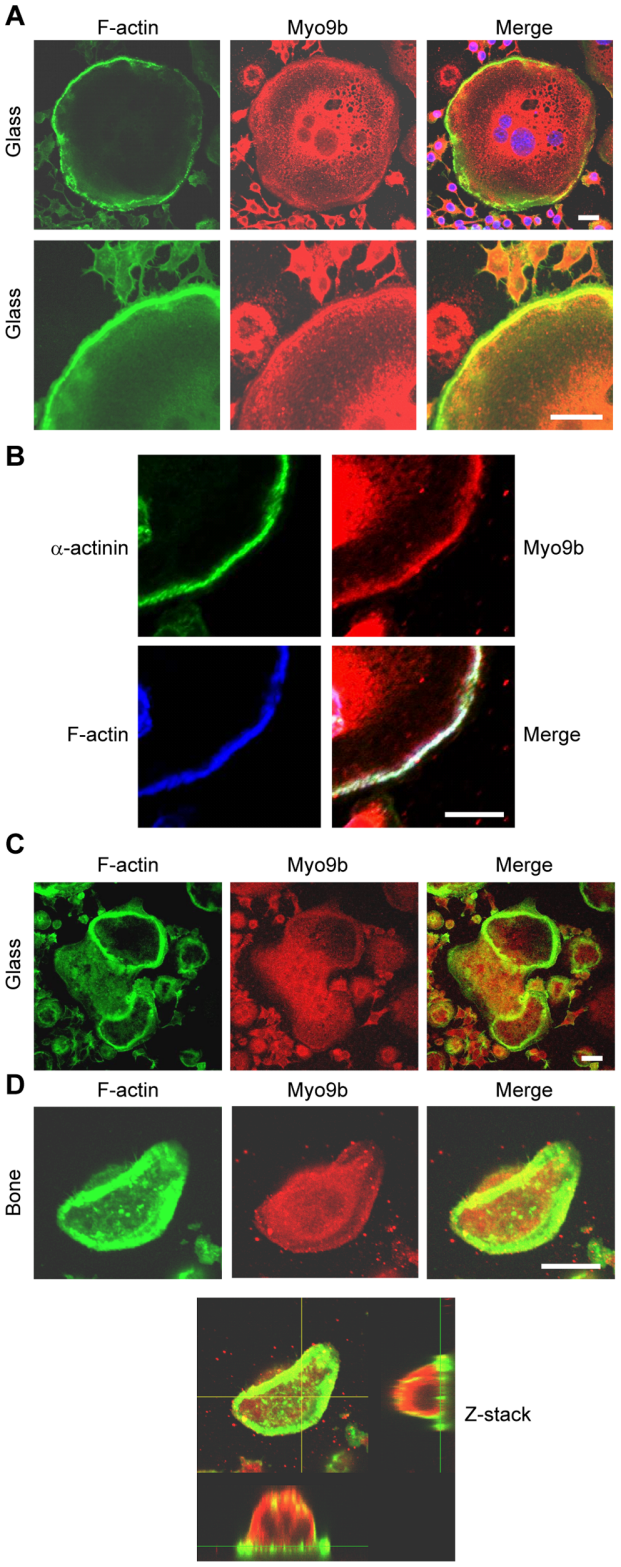
Distribution of Myo9b in osteoclasts. Osteoclast podosomes and sealing zones were labeled with fluorescent phalloidin and anti-Myo9b antibodies and viewed by confocal microscopy. *A*, In mature osteoclasts on glass coverslips, Myo9b is at its highest levels in the perinuclear region and in the peripheral podosome belt. Nuclei are visible in blue in the merged image. *B*, In osteoclasts on glass coverslips, Myo9b associates closely with the podosome core protein α-actinin. *C*, In immature osteoclasts on glass that have not formed peripheral podosome belts, Myo9b is present, though not enriched, in internal podosome rings. *D*, Myo9b is mostly absent from sealing zones in osteoclasts on bone. A Z-stack image of an osteoclast on bone demonstrates that Myo9b is present throughout the cytoplasm. Scale bars  = 20 µm.

To determine the intracellular distribution of Myo9b in osteoclasts, cells were labeled by immunocytochemistry and examined by confocal microscopy. Figure 1A demonstrates that in cells on glass coverslips, Myo9b was somewhat diffuse throughout the cell but was enriched both in the perinuclear region and in the peripheral podosome belt visible by F-actin labeling. Myo9b was particularly well localized to regions where the belt adopted an uninterrupted arrangement of podosomes at the extreme periphery, suggesting maturity of podosome organization [26]. Co-labeling of osteoclasts for F-actin and the podosome core protein α-actinin [44] demonstrated Myo9b's association with the podosome belt (Figure 1B). However, in immature osteoclasts that did not form peripheral podosome belts but instead formed internal podosome rings, Myo9b was present in these structures but not enriched (Figure 1C). Because the Myo9b RhoGAP domain is inhibitory to Rho signaling, this finding is consistent with previous reports demonstrating that high Rho activity produces internal podosome rings in cells on glass, while low Rho activity produces peripheral belts [32], [34]. Similarly, when osteoclasts were on bone, Myo9b was nearly absent from sealing zones, which are structures produced by high Rho activity [34]. Instead, this myosin appeared to be diffusely present throughout the cytoplasm (Figure 1D). Myo9a expression was undetectable in osteoclasts by RT-PCR (data not shown), consistent with the finding that it is primarily a brain and testis-restricted form [5].

### RNAi-mediated knockdown of Myo9b increases Rho activity in osteoclasts

**Figure 2:**
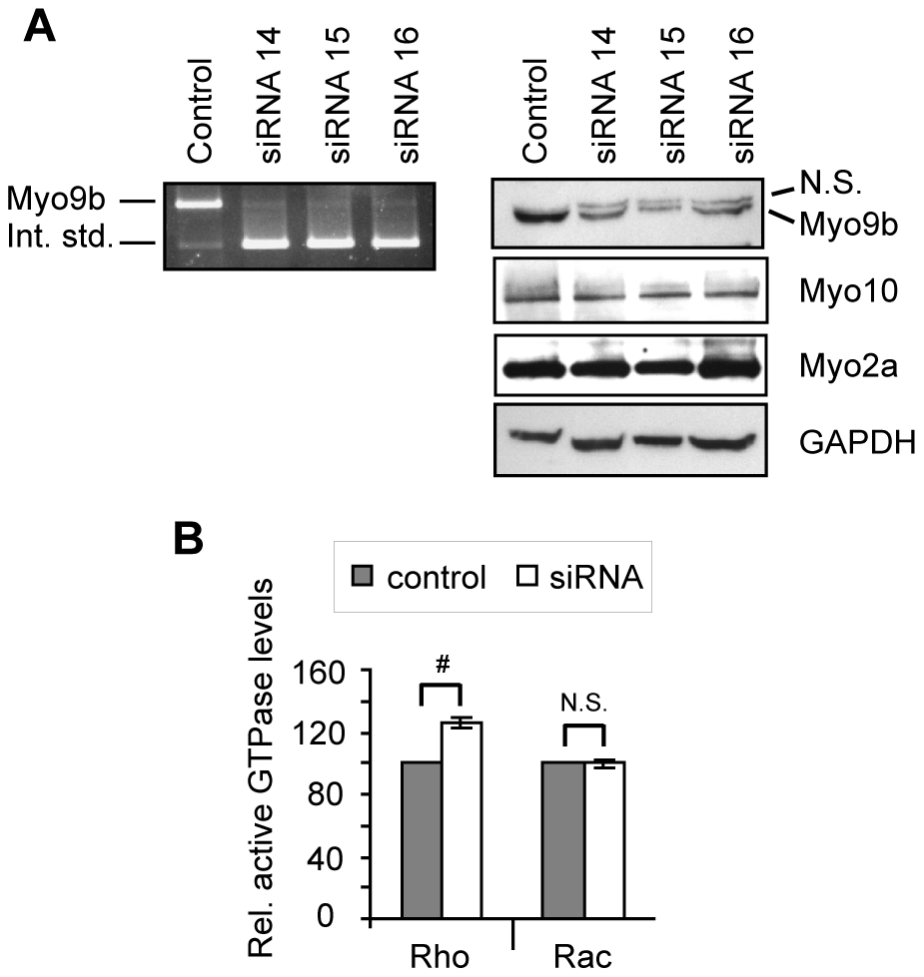
Knockdown of Myo9b increases cellular Rho activity. *A*, Competitive RT-PCR (left panel) and Western blot (right panels) show efficient knockdown of Myo9b in mouse bone marrow-derived osteoclasts. For competitive RT-PCR, 1 pg of a synthetic RNA containing the Myo9b primer binding sites (the internal standard) was added to 1 µg sample total RNA, as described in Materials and Methods. RT-PCR then resulted in amplification of both cellular Myo9b and the internal standard, which served as a control for relative Myo9b mRNA levels. By Western analysis, knockdown of Myo9b did not significantly change protein expression of two other osteoclast myosins. GAPDH is shown as a loading control. N.S.  =  non-specific band. *B*, Protein pull-down followed by Western analysis and densitometry was used to quantify levels of active Rho or Rac in control or Myo9b siRNA-treated marrow-derived osteoclasts. Levels of active small G-proteins were normalized to levels of total Rho or Rac. The graph was compiled from three such experiments and shows that knockdown of Myo9b resulted in increased cellular levels of Rho but not Rac. #: *P*<0.001; N.S.: not significant.

To determine the function of Myo9b, its expression was knocked down in osteoclasts derived from mouse bone marrow (MBM) by transfection of siRNAs. Quantitative real-time RT-PCR revealed average knockdown of these three siRNAs at 95±7% (n = 4). This result was confirmed by a gel-based competitive RT-PCR method in which Myo9b mRNA was reverse transcribed and amplified in the presence of a synthetic internal standard RNA that was reverse transcribed and amplified by the same primers. As shown in Figure 2A (left panel), siRNA treatment led to nearly undetectable levels of Myo9b mRNA. Western analysis of Myo9b revealed a close doublet of bands. The lower band was intense in control-transfected cells, with the upper band much less visible. In three separate experiments, knockdown of Myo9b resulted in about a 50% loss of signal intensity in the lower band while the upper band did not diminish (Figure 2A, right). Based on these findings, it is believed that the upper band represents non-specific labeling by the antibody. The relative lack of protein suppression relative to mRNA suppression with siRNA treatment is typical of other osteoclast myosins [41], [43] and is likely due to the extreme stability of these proteins. For example, we demonstrated that another osteoclast myosin, Myo10, has a half-life of nearly 5 days [41]. Osteoclasts express multiple myosin isoforms ([40], [41], [43] and our unpublished data), and to determine whether expression of others may be affected by Myo9b knockdown, control and siRNA-treated protein lysates were probed for Myo10, a myosin with a key role in sealing zone patterning [41], and for Myo2a, which acts as a negative regulator of pre-osteoclast fusion [40], [43]. There was little to no change in expression of these proteins (Figure 2A).

To determine the role of Myo9b in regulating Rho family GTPases, levels of total and active Rho were measured in both control and siRNA-treated osteoclasts. Knockdown of Myo9b increased active Rho levels by 26±3% without affecting levels of active Rac (Figure 2B). Similar results were obtained in RAW264.7-derived osteoclasts, as ∼50% knockdown in these cells also resulted in a 36±7% increase in active Rho levels without affecting Rac activity (not shown). While many RhoGAPs inactivate multiple small GTPases, this finding is consistent with previous reports demonstrating that Myo9b's GAP activity is specific for the Rho subfamily, particularly RhoA [5], [20], [21]. However, the magnitude of increase is notable, as mammalian genomes are predicted to express dozens of RhoGAPs [45]. The results shown here indicate that Myo9b is a major regulator of Rho signaling in osteoclasts.

### Suppression of Myo9b levels leads to altered podosome patterning and decreased directional motility

**Figure 3:**
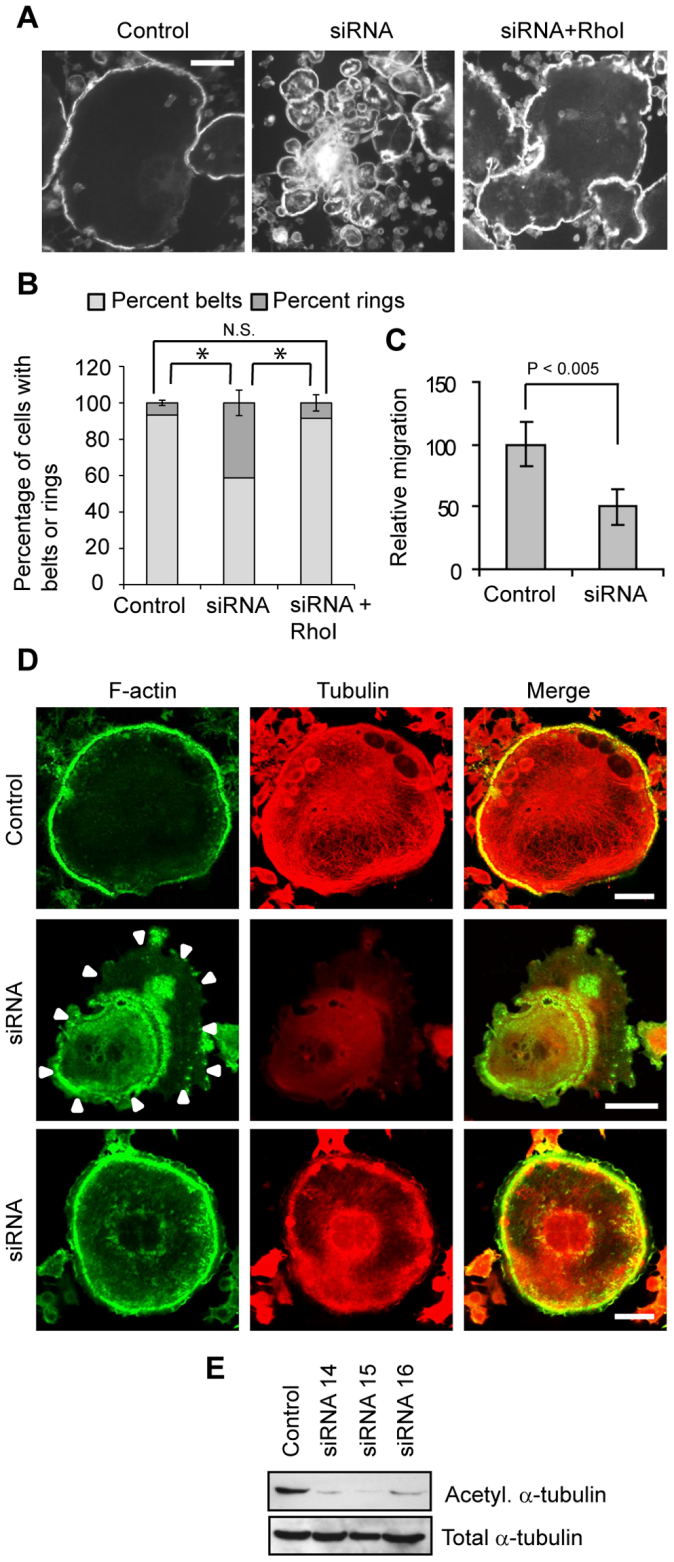
Knockdown of Myo9b causes Rho-dependent changes in podosome patterning and microtubule stability. *A*, Fluorescent phalloidin labeling demonstrates that suppression of Myo9b results in loss of podosome belt formation that is reversed by inhibition of Rho with a cell-permeant C3 transferase. Scale bars  = 20 µm. *B*, Suppression of Myo9b decreases the percentage of mature osteoclasts with podosome belts in a Rho-dependent manner. *: *P*<0.001; N.S.  =  not significant. *C*, Knockdown of Myo9b significantly decreases osteopontin-directed motility of marrow-derived osteoclasts. *D*, Knockdown of Myo9b causes loss of microtubule networks in the majority of siRNA-treated osteoclasts. Arrowheads indicate the cell periphery in an siRNA-treated cell. Scale bars  = 20 µm. *E*, Suppression of Myo9b by siRNAs strongly diminishes acetylation of α-tubulin in osteoclasts while not affecting its total expression.

To determine whether Myo9b plays a role in osteoclast function, osteoclasts with siRNA-suppressed Myo9b levels were first assessed for morphological changes. Control or siRNA-treated osteoclasts on glass were labeled with fluorescent phalloidin to reveal podosome patterning. Figure 3A demonstrates that while mature control osteoclasts formed podosome belts at the cell periphery, osteoclasts with suppressed levels of Myo9b formed fewer peripheral belts but more clusters of internal rings and F-actin patches. This phenomenon was reversed when siRNA-treated cells were treated with an inhibitor of Rho (exoenzyme C3 transferase), demonstrating that the RhoGAP activity of Myo9b, rather than activity of motor or other domains, was primarily responsible for the effect. The percentages of cells displaying the belt or ring phenotype under each condition are indicated graphically in Figure 3B. Not unexpectedly, the aberrant podosome patterning was associated with a decreased ability of cells to migrate in a directional manner (Figure 3C). Previous studies have shown that the ability of osteoclasts to form peripheral podosome belts is a microtubule-dependent process. Indeed, when microtubules are disrupted or their association with podosomes is inhibited, osteoclasts form small internal actin rings similar to those described here [26], [41]. Therefore, microtubule architecture in Myo9b-suppressed osteoclasts was assessed by immunocytochemistry. As shown in Figure 3D, all control-transfected osteoclasts demonstrated a radial microtubule network that spread throughout the cell and extended to the cell periphery, as is well established for wild-type murine osteoclasts. In contrast, a large percentage of siRNA-treated cells (73±7%) demonstrated a collapse of this network, with only diffuse tubulin staining and a lack of microtubules extending to the cell periphery. Of the siRNA-treated cells lacking a peripheral podosome belt, nearly all (99±2%) lacked a robust microtubule network (Figure 3D, middle row). However, 59±6% of the siRNA-treated cells that displayed podosome belts also were deficient in microtubules (Figure 3D, bottom row). This finding was surprising given the critical role of microtubules in promoting podosome belt formation. In these experiments, it is possible that microtubules were able to form and drive podosome patterning, but did not remain stable following belt formation. The levels of microtubule acetylation in these cells were then assessed, as acetylation of α-tubulin is a marker of microtubule stability and is in fact inversely related to Rho activity [32]. As shown in Figure 3E, suppression of Myo9b by any of three siRNAs markedly reduced acetylation of microtubules without affecting α-tubulin expression. These findings together reveal a role for the Rho inhibitory activity of Myo9b in generating stable microtubules, and consequently, podosome patterning.

### Suppression of Myo9b levels dramatically reduces resorptive activity although sealing zone morphology is unaffected

**Figure 4:**
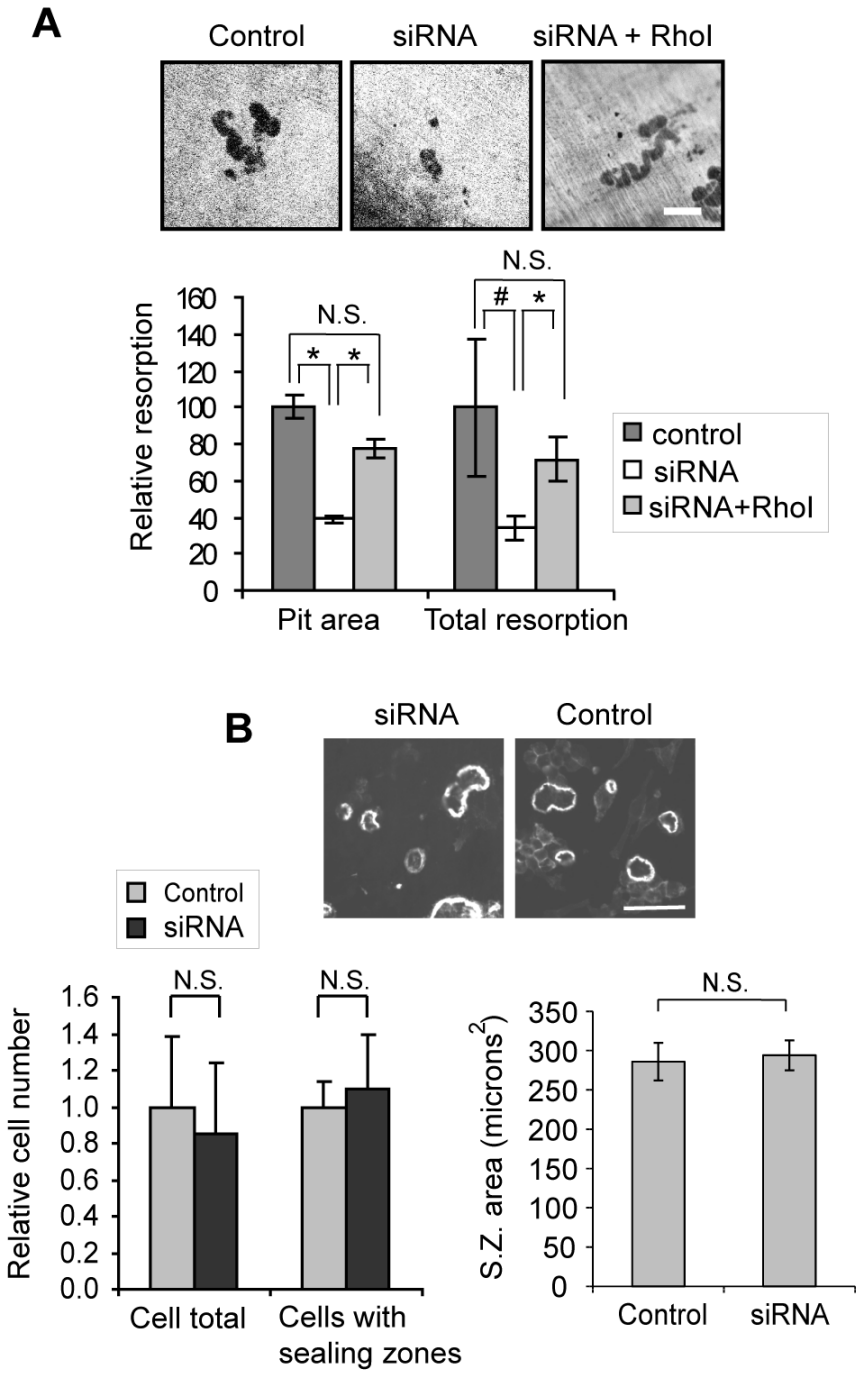
Knockdown of Myo9b causes a Rho-dependent loss of bone resorptive capacity. *A*, siRNA-mediated knockdown of Myo9b diminishes resorptive capacity of osteoclasts in a Rho-dependent manner, as demonstrated by photomicrographs of resorption pits on ivory slices (top). Scale bar  = 50 µm. Quantification of the surface area of each pit (pit area) and total resorbed surface area (total resorption) are shown graphically below. #: *P*<0.05; *: *P*<0.005; N.S.  =  not significant. *B*, Knockdown of Myo9b does not significantly affect osteoclast number or the ability of cells to form sealing zones on bone, as indicated by photomicrographs of phalloidin-labeled sealing zones (top) and enumeration of cell number and sealing zone number and size (bottom).

Alterations in podosome patterning produced by suppression of Myo9b suggest that sealing zone formation and resorptive capacity of osteoclasts may also be regulated by this myosin. Therefore, control or siRNA-treated MBM-derived osteoclasts were tested for their ability to resorb bone. Figure 4A demonstrates that osteoclasts subjected to Myo9b knockdown showed a significantly decreased ability to resorb, as displayed by photomicrographs of typical resorption pits and graphically by decreased average pit area and overall resorption. Similar results were obtained with cells cultured on synthetic substrate (not shown). Further, inhibition of Rho activity was able to reverse this effect, again indicating that excessive Rho signaling caused by knockdown of Myo9b RhoGAP activity has a deleterious effect on osteoclast function (Figure 4A). One way in which loss of Myo9b might decrease resorption is through affecting formation or size of sealing zones or altering osteoclast number. However, sealing zone morphology, size, and number were unaffected by the knockdown, as shown by phalloidin-labeled images and quantification of these attributes (Figure 4B). Total cell number was also unaffected and no differences in the rates of apoptosis were observed (not shown). Therefore, the reduction in resorptive capacity induced by Myo9b knockdown was not due to cell death or altered sealing zone morphology, but rather by a Rho-dependent process that interferes with the signaling mechanisms that trigger resorption.

**Figure 5:**
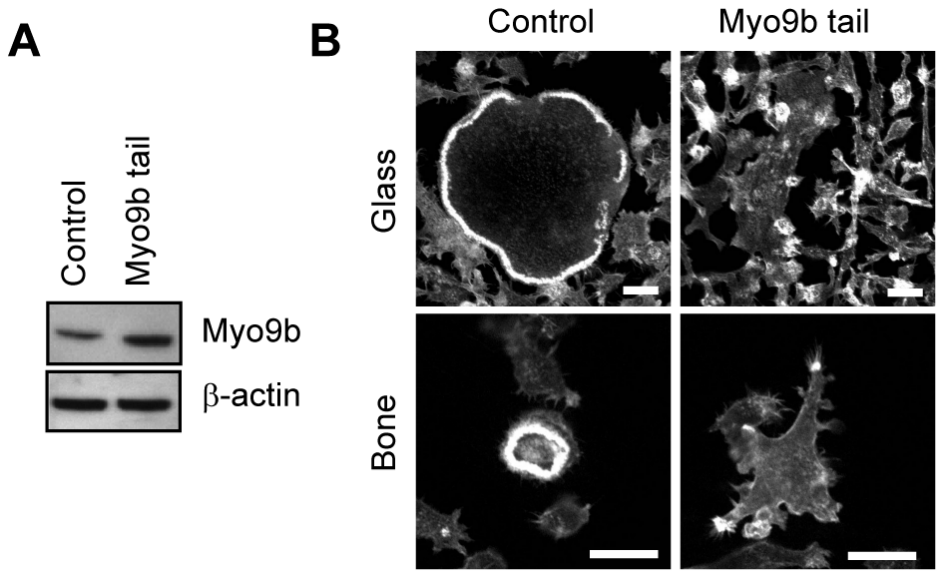
Overexpression of the Myo9b tail domain alters podosome and sealing zone patterning. *A,* Western analysis demonstrates that transfected osteoclasts express levels of the Myo9b tail that are about 2-fold greater than control (transfected with empty vector). *B*, Fluorescent F-actin labeling of osteoclasts generated from control or Myo9b-transfected cells shows that F-actin distribution and spreading are severely disrupted in cells plated either on glass or bone. Scale bars  = 20 µm.

The presence of RhoGAP domains in myosin molecules dictates that these domains are associated with a specific pool of cellular actin filaments. To determine how dissociation of the RhoGAP domain from these filaments might affect cytoskeletal dynamics, we stably overexpressed the Myo9b tail in RAW264.7 macrophages. Even with the use of a non-robust cellular promoter, this treatment was toxic to cells, as only a single clone survived selection. However, this clone, which expressed the Myo9b tail at about 2-fold normal levels, showed a marked inability to spread properly. Further, podosome belts and sealing zones were not formed in any of these cells, but instead small patches or clusters of F-actin were generated either on glass or ivory substrates (Figure 5). These results demonstrate that targeting of the Myo9b RhoGAP moiety to specific F-actin pools through association of the myosin head is required for normal function.

**Figure 6:**
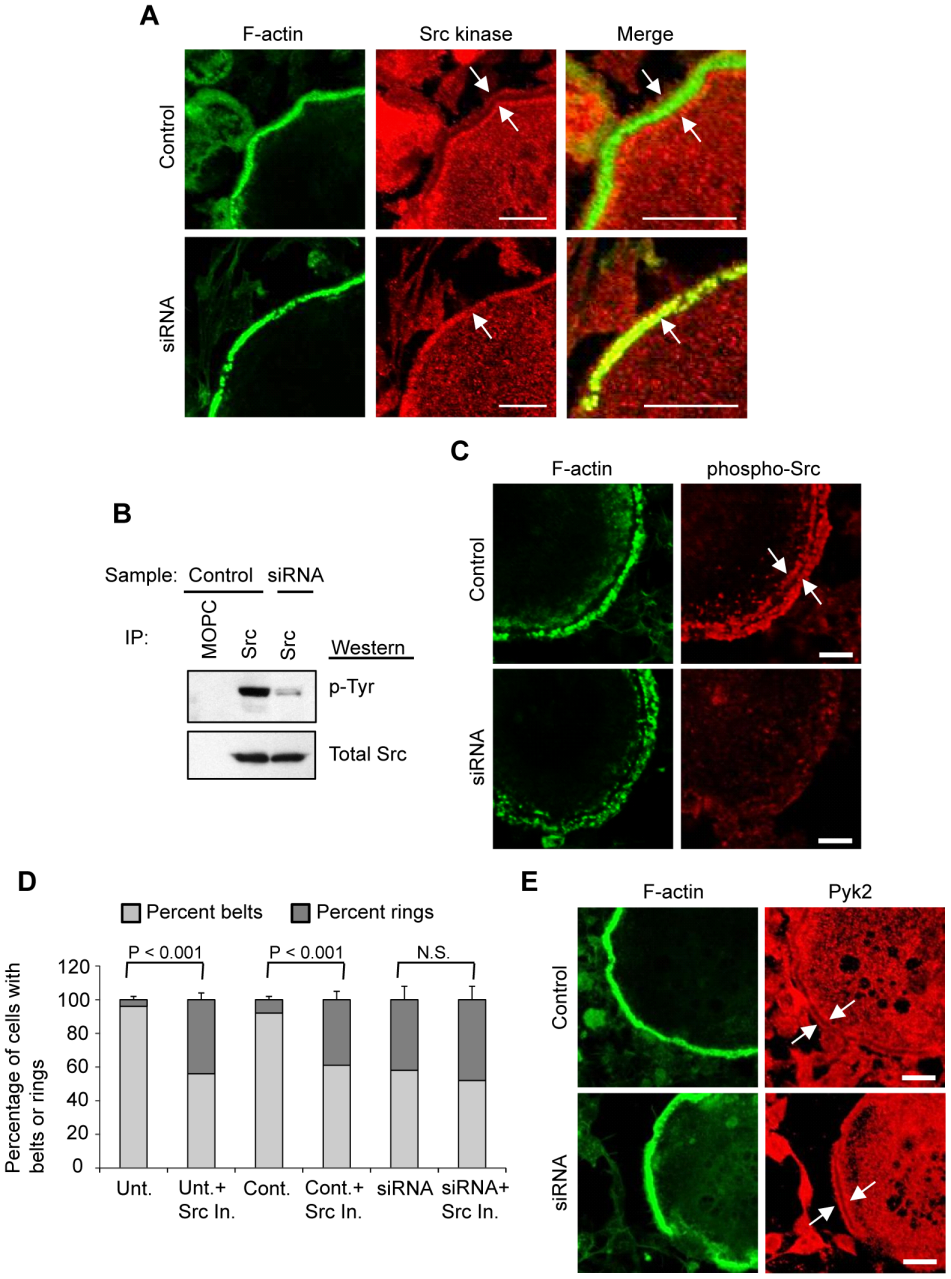
Knockdown of Myo9b causes mislocalization and diminished activation of Src kinase. *A*, Immunocytochemical labeling of podosome belts in mature osteoclasts demonstrates that Src is mislocalized when Myo9b levels are suppressed. Arrows indicate the normal “railroad track” distribution of Src around podosome belts in control cells, while Src is present in the F-actin core of podosomes in siRNA-treated cells. *B*, Immunoprecipitation of total cellular Src kinase followed by Western blot with a phospho-tyrosine antibody demonstrates that siRNA treatment diminishes Src phosphorylation without affecting Src protein levels. One lane of immunoprecipitation with the mouse IgG MOPC-21 was added as a negative control. *C*, Immunocytochemical labeling of osteoclasts with an antibody against phosphorylated Src shows its relative absence in siRNA-treated cells. *D*, Inhibition of Src kinase causes loss of podosome belts in untreated and control osteoclasts, but does not further diminish podosome belts in cells with suppressed levels of Myo9b. *E*, Immunocytochemical analysis shows that Pyk2 distribution does not change with siRNA treatment. For all photomicrographs in this figure, scale bars  = 20 µm.

One way in which the lowered Myo9b levels produced by siRNA treatment might impact osteoclast function is through mislocalization of various actin modulating or signaling proteins involved in osteoclast cytoskeletal arrangements. Notably, the distribution of Src kinase was found to be significantly altered in Myo9b siRNA-treated cells. In control osteoclasts, Src adopted a “railroad track” pattern around podosome belts but was absent from the podosome actin core. However, in a large percentage of siRNA-treated cells containing podosome belts (56±7%), Src directly overlapped this podosome core (Figure 6A). Mislocalization of Src is particularly notable as it is essential for proper osteoclast actin organization and resorptive capacity [46], [47]. Activation (tyrosine phosphorylation) of Src was also inhibited, as determined both by immunoprecipitation of Src followed by Western blot for phosphotyrosines (Figure 6B) and immunocytochemical labeling of phosphorylated Src (Figure 6C). In addition, inhibition of Src in untreated and control-transfected osteoclasts resulted in a loss of podosome belts and formation of internal rings similar to that caused by knockdown of Myo9b (Figure 6D). Inhibition of Src did not result in a loss of podosome belts beyond that caused by Myo9b knockdown (Figure 6D). Finally, immunocytochemistry was performed using antibodies against various proteins known to associate with podosomal/sealing zone complexes. We saw no differences between control and siRNA-treated cells in distribution of α-actinin and cortactin, two proteins that are present in the actin core of podosomes (not shown). Nor were differences observed between control and siRNA-treated cells in the distributions of Pyk2, vinculin, and paxillin, podosome proteins that co-localize with Src at the periphery of podosomes [25] (Figure 6E and data not shown). Because recruitment of Src to tyrosine kinase Pyk2 is required for activation of Src and downstream cytoskeletal rearrangements [48], [49], it is likely that its mislocalization relative to Pyk2 and other podosomal proteins is responsible for the lack of Src activation following knockdown of Myo9b.

**Figure 7:**
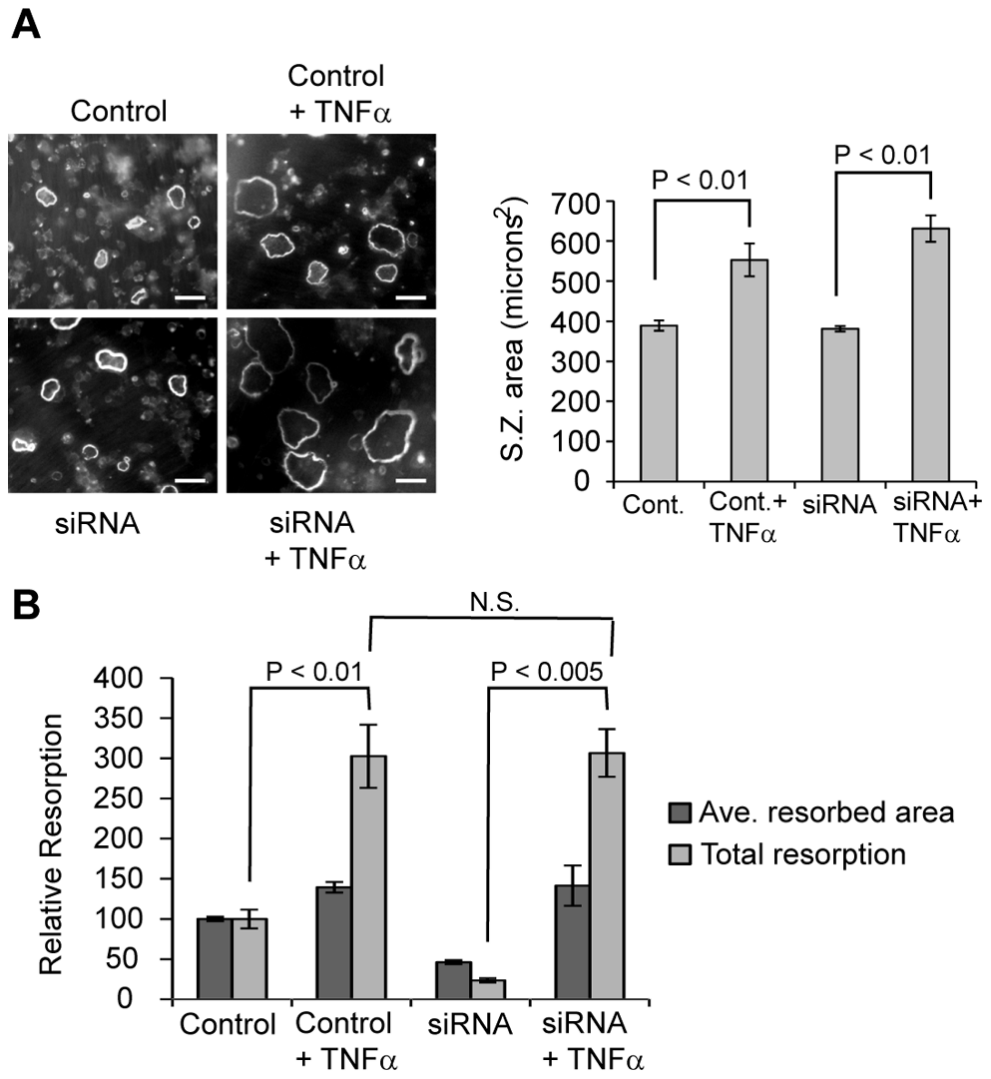
TNFα rescues osteoclast defects caused by loss of Myo9b expression. *A*, Overnight addition of TNFα to mature control or siRNA-treated osteoclasts results in larger sealing zones, as shown by phalloidin labeling of cells on bone (left) and quantification of sealing zone perimeter (right). Scale bars  = 100 µm. *B*, Overnight addition of TNFα to mature or siRNA-treated osteoclasts increases the average size of individual resorbed areas and strongly increases levels of total resorption.

As described above, loss of Myo9b function has been suggested to play a role in impaired integrity of the intestine, potentially contributing to inflammatory diseases of the bowel [13]. Common extra-intestinal manifestations of these diseases are osteopenia and osteoporosis, due in part to excessive bone resorption. The relatively inactive osteoclasts produced by loss of Myo9b function in this study would seem to be inconsistent with these findings. However, TNFα, an inflammatory cytokine with a critical role in the pathogenesis of the inflammatory bowel diseases [50], [51], has previously been shown to alter cellular morphology through its interaction with Rho signaling pathways [52]. TNFα also has been demonstrated to promote sealing zone formation and bone resorption in mature osteoclasts [53]. Therefore, mature cells were treated overnight with high levels of TNFα (10 ng/ml), and cellular morphology and resorptive capacity were assessed. Figure 7A demonstrates that addition of TNFα to control osteoclasts produced an increase in sealing zone area. Accordingly, this treatment also resulted in an enhancement of resorptive capacity over control levels (Figure 7B). Notably, TNFα treatment restored the defect in osteoclast resorption produced by Myo9b knockdown. SiRNA-treated osteoclasts cultured in the presence of TNFα displayed increased sealing zone area similar to that of control cells cultured with TNFα (Figure 7A). In addition, siRNA-treated cells cultured in TNFα were able to resorb at the same levels as control cells that underwent TNFα treatment (Figure 7B). These results indicate that high levels of this cytokine are capable of overcoming the defective cytoskeletal organization of osteoclasts with suppressed Myo9b levels. These results further suggest that the loss of function produced by suppression of Myo9b may be reversed in an inflammatory environment.

## Discussion

The Rho family of small GTPases plays a critical role in osteoclast function. Indeed, an important class of therapeutics for inhibiting excessive osteoclast activity, the nitrogen-containing bisphosphonates, works to inhibit Rho function by preventing prenylation of these small GTPases, which is necessary for their membrane association and activation [54]. In particular, RhoA is a key regulator of the actin-rich sealing zone, a substrate adhesion structure that is required for generating the resorptive capacity of osteoclasts. While numerous studies have elucidated the role of RhoA in osteoclasts, little is known about the regulatory proteins that directly modulate its function, the RhoGAPs and RhoGEFs (Rho guanine nucleotide exchange factors, activators of Rho function). A survey in RAW264.7-derived osteoclasts identified expression of 42 of the 76 known murine RhoGEFs in these cells, and further identified two that were upregulated during and necessary for osteoclast differentiation [55]. In contrast, we are unaware of any surveys of RhoGAPs in osteoclasts, although up to 70 have been predicted to be expressed in mammalian genomes [45]. Here we have demonstrated that a knockdown of the RhoGAP Myo9b by only about 50% was sufficient to increase Rho activity by approximately 30%, indicating that Myo9b is a major contributor to overall Rho activity in osteoclasts. The results presented here also demonstrate that regulation of Rho at a local, rather than cellular, level is likely to be key to osteoclast cytoskeletal dynamics. Immunocytochemical analysis demonstrated that Myo9b associates strongly with podosomes, structures known to form in the absence of Rho activity, while associating less well with internal actin rings and sealing zones on bone, which are formed under conditions of high Rho activity. Further, transfection of the Myo9b tail, which contains the RhoGAP domain but no head domain to associate with actin, caused massive disruption of cell adhesion structures. In spite of the fact that Myo9b appears to be a dominant regulator of Rho signaling in osteoclasts due to its effects on total cellular Rho activity, its targeting to appropriate subcellular domains is critical to exerting the appropriate effects on the cytoskeleton.

Classic assessments of Rho activation in cellular functions have relied on tools that measure or alter Rho activity on a whole-cell level, including the use of exoenzyme C3 transferase and Rho pull-down assays as shown here, along with the overexpression of dominant-positive and dominant-negative Rho mutants. However, with the utilization over the last decade of fluorescent biosensors for Rho activity in living cells, it has become clear that Rho, like other related small GTPases, exerts its effects on specific subcellular domains in a regulated spatio-temporal manner (rev. in [56]). It is likely that each subcellular location is regulated by different upstream effectors such as the RhoGAPs and RhoGEFs. Here we suggest that Myo9b, by virtue of its segregation to podosome belts and its lack in sealing zones, regulates local Rho signaling to generate these structures. The question still remains as to how Myo9b distribution is regulated. One possibility is that isoforms of the actin-regulatory protein tropomyosin may play a role in this distribution. Tropomyosins bind and control access to actin filaments by other actin regulatory proteins, including myosins. They also have been suggested to act as local interpreters of Rho signaling since several isoforms were shown to stimulate actin structures that mimic the effects of Rho GTPase signaling pathways [57]. In osteoclasts, at least seven isoforms are expressed and these localize to discrete subcellular domains, including the specialized adhesion structures [38]. It is imaginable that one or more of these tropomyosin isoforms may control Myo9b binding to various F-actin subcellular domains, but this possibility requires further study.

We also found that Myo9b knockdown caused a lack of bone resorptive capacity and that this was due to excessive Rho activation, as addition of a Rho inhibitor reversed this effect. This finding was someone unexpected, since Rho activation has been well established to play a key role in promoting osteoclastic resorptive activity. However, other studies have demonstrated an inverse relationship between Rho activity and microtubule stability [32], [58], and have shown that excessive Rho activity in osteoclasts results in microtubule destabilization and impaired bone resorption [58]. Therefore, this study confirms that Rho activity can indeed be too much of a good thing for optimal cell function, at least as localized to osteoclast adhesion structures. This is also consistent with the finding that macrophages obtained from Myo9b knockout mice showed Rho-dependent defects in function, including motility [14]. Similar to our osteoclasts, these macrophages were unable to properly spread or polarize into a migratory morphology and had severely impaired responses to a chemoattractant gradient. Inhibition of Rho reversed these effects although the macrophages could not properly retract their tails during migration. This impaired migration was reflected *in vivo*, where Myo9b knockout mice showed a severe reduction of monocytes and macrophages recruited to the peritoneal cavity using an experimental model of C5a-induced peritonitis. Based on these findings and our own, it is tempting to speculate that recruitment of monocytic osteoclast precursors to bone and subsequent function of mature osteoclasts may also be impaired in these animals. Indeed, our findings suggest that loss of Myo9b function would produce generally ineffective osteoclasts that could nonetheless be stimulated to excessive activity by an inflammatory environment. *MYO9B^-/-^* mice are smaller than wild-type littermates from a very early age, which could be indicative of skeletal impairment caused by cellular defects ([14] and our data). We have begun direct study of the skeletons of these mice to determine whether loss of Myo9b expression specifically impairs osteoclast precursor recruitment or osteoclast function *in vivo*.

The mislocalization and poor activation of Src kinase observed following Myo9b knockdown is highly likely to play a key role in the impaired motility and resorptive capacity of these cells. Src is necessary for these functions in osteoclasts; indeed, although Src is widely expressed in mammalian cells, the primary phenotype of its knockout in mice is osteopetrosis, a condition of thickened, brittle bones caused by failure of osteoclasts to resorb bone [46]. Mislocalization of tight junction-associated proteins was also observed in intestinal epithelial cells following knockdown of Myo9b [13], confirming that Myo9b is critical for generating an appropriate distribution of membrane-associated proteins involved in multiple cellular functions. The mechanism by which Myo9b-mediated Rho activity alters Src distribution is unclear. However, likely candidates for mediating this effect are the formin homology proteins mDia1-3 [59]. mDia proteins are regulators of the cytoskeleton that mediate downstream effects of Rho GTPases, including RhoA, which is known to be critical for osteoclast cytoskeletal dynamics. Notably, these proteins also interact with Src and link it to Rho function. For example, it is established that Rho-mDia1 signaling can mobilize Src to focal adhesions, regulating cell polarity and motility [60]. However, little is known about mDia1 function in osteoclasts. In contrast, mDia2 was shown to have a clear effect on osteoclast podosome patterning through regulation of tubulin acetylation [32].

Post-translational modification of tubulin is a powerful means by which microtubule architecture is modified [61]. While acetylation of microtubules is a marker for their stability, it is yet unclear whether the acetylation directly promotes this stability. In osteoclasts, expansion of internal actin rings into peripheral podosome belts is a microtubule-dependent process, and acetylation of microtubules is associated with formation of peripheral podosome belts and bone resorptive capacity [26], [32]. Rho activity, which is heightened in Myo9b-suppressed cells, is inversely correlated with microtubule stability in osteoclasts [32], [58]. This is due at least in part to Rho-mediated activation of the formin homology protein mDia2, which then activates the tubulin deacetylase HDAC6, resulting in altered podosome patterning. This finding is consistent with the work presented here demonstrating that knockdown of Myo9b and subsequent increased Rho levels results in a loss of microtubule acetylation. Thus, mDia2 may play a role in the signaling pathways described here, but this hypothesis requires more extensive study. It is yet unclear why a large percentage of Myo9b siRNA-treated cells in this study were capable of forming podosome belts in the apparent absence of a robust microtubule network. Because fixed cells were used, we were unable to determine whether microtubule networks drove podosome belt formation and then dissociated, or whether podosome belts were formed in the absence of microtubules. We suspect the former scenario to be more likely, but imaging of living cells will be required to answer this question more fully.

Interestingly, osteoclasts from Pyk2-deficient mice exhibit many of the same deficits as Myo9b-suppressed osteoclasts [58]. Pyk2, a tyrosine kinase necessary for osteoclast function, is required for proper recruitment of Src to podosomes. *A priori*, cells lacking Pyk2 might be expected to display similarities to the cells in this study that also demonstrate poor recruitment of Src, and this is indeed the case. Similar to Myo9b siRNA-treated cells, *Pyk2^-/-^* osteoclasts display decreased resorptive capacity, reduced Src activation, excessive Rho activity, impaired tubulin acetylation and lack of a normal microtubule network, formation of small actin rings instead of podosome belts, and normal distributions of cortactin, vinculin and paxillin. However, unlike the cells in this study, *Pyk2^-/-^* osteoclasts also displayed sealing zones that were smaller and thinner than normal, while Myo9b-suppressed sealing zones were of normal shape and size. This difference in phenotype may be due to our finding that Myo9b is not a normal component of sealing zones, and therefore its knockdown does not affect their overall shape. Nonetheless, these results are highly consistent with the characteristics of Myo9b-suppressed cells that result from an inability of Src to be appropriately distributed and activated in podosome belts.

Our previous work has demonstrated distinct roles for other classes of myosins in osteoclasts. Myosin IIA (Myo2a) was shown to be required for proper osteoclast spreading, motility, and resorptive capacity, but perhaps more notably, its expression was demonstrated to be post-translationally modulated during differentiation to regulate fusion of osteoclast precursors [43]. In contrast, Myo10, the sole member of class X myosins, was shown to directly link osteoclast podosomes and microtubule networks to promote maturation of podosome belts and sealing zones [41]. Osteoclasts, by virtue of their large size and dynamic actin cytoskeletons, have been a useful model for elucidating some of the cellular functions of these varied myosin classes. Given the association of Myo9b with known inflammatory diseases, it will be of interest to determine how mutations and loss of its expression in osteoclasts affect bone quality *in vivo*.
